# HIIT'ing or MISS'ing the Optimal Management of Polycystic Ovary Syndrome: A Systematic Review and Meta-Analysis of High- Versus Moderate-Intensity Exercise Prescription

**DOI:** 10.3389/fphys.2021.715881

**Published:** 2021-08-16

**Authors:** Cory T. Richards, Victoria L. Meah, Philip E. James, D. Aled Rees, Rachel N. Lord

**Affiliations:** ^1^School of Sport and Health Sciences, Cardiff Metropolitan University, Cardiff, United Kingdom; ^2^Program for Pregnancy and Postpartum Health, Faculty of Kinesiology, Sport, and Recreation, Women and Children's Health Research Institute, Alberta Diabetes Institute, University of Alberta, Edmonton, AB, Canada; ^3^Neuroscience and Mental Health Research Institute, School of Medicine, Cardiff University, Cardiff, United Kingdom

**Keywords:** PCOS, exercise, moderate-intensity, high-intensity, insulin resistance, cardiorespiratory fitness, cardiometabolic risk

## Abstract

**Introduction:** Polycystic Ovary syndrome (PCOS) is a metabolic disorder associated with increased cardiovascular disease risk. Exercise is an effective treatment strategy to manage symptoms and reduce long-term health risk. High-intensity interval training (HIIT) has been suggested as a more efficient exercise mode in PCOS; however, it is not clear whether HIIT is superior to moderate intensity steady state exercise (MISS).

**Methods:** We synthesized available data through a systematic review and meta-analysis to compare the effectiveness of isolated HIIT and MISS exercise interventions. Our primary outcome measures were cardiorespiratory fitness and insulin resistance, measured using $\dot{\text{V}}$O_2max_ and HOMA-IR respectively.

**Results:** A total of 16 studies were included. Moderate-quality evidence from 16 studies identified significant improvements in $\dot{\text{V}}$O_2max_ following MISS (Δ = 1.081 ml/kg/min, *p* < 0.001, *n* = 194), but not HIIT (Δ = 0.641 ml/kg/min, *p* = 0.128, *n* = 28). Neither HIIT nor MISS improved HOMA-IR [(Δ = −0.257, *p* = 0.374, *n* = 60) and (Δ = −0.341, *p* = 0.078, *n* = 159), respectively].

**Discussion:** A significant improvement in $\dot{\text{V}}$O_2max_ was evident following MISS, but not HIIT exercise in women with PCOS. This contrasts with previous literature in healthy and clinical cohorts that report superior benefits of HIIT. Therefore, based on available moderate-quality evidence, HIIT exercise does not provide superior outcomes in $\dot{\text{V}}$O_2max_ compared with MISS, although larger high-quality interventions are needed to fully address this. Additional dietary/pharmacological interventions may be required in conjunction with exercise to improve insulin sensitivity.

## Introduction

Polycystic Ovary syndrome (PCOS) is the most common endocrine condition, affecting between 5 and 21% of the premenopausal population (Teede et al., 2010; Azziz et al., 2016; Lizneva et al., 2016), and is the leading cause of anovulatory infertility (Moran et al., 2017). Criteria for diagnosis include 2 or more of: biochemical or clinical hyperandrogenism, irregular or absent menses, and the presence of morphological polycystic ovaries (Rotterdam ESHRE/ASRM-Sponsored PCOS Consensus Workshop Group, 2004). In addition to its reproductive sequelae, PCOS is recognized as a metabolic disorder that increases the prevalence of cardiovascular risk factors including hypertension and type 2 diabetes mellitus (Kakoly et al., 2019), which may increase the likelihood of developing cardiovascular disease (CVD) (Talbott et al., 2004; Teede et al., 2018; Berni et al., 2021). A key alteration in PCOS is insulin resistance (IR), which is central to disease pathogenesis and intrinsic to the condition (Cassar et al., 2016). The intrinsic IR experienced by women with PCOS has the potential to exacerbate or be affected by risk factors such as obesity (Cassar et al., 2016) and hyperandrogenism (Burghen et al., 1980; Diamanti-Kandarakis and Dunaif, 2012).

Management decisions are driven by symptomatic need. Lifestyle and diet modification, and pharmacological interventions are commonly utilized. However, adherence to treatment interventions, including lifestyle and pharmacological methods, is often poor in this population, and has been reported as low as 21% (Hoeger, 2008; Kim et al., 2020; Parker et al., 2020). Exercise, alone and in conjunction with concurrent interventions, has recently been reviewed (dos Santos et al., 2020; Patten et al., 2020). Studies of moderate-intensity steady state (MISS) exercise prescription in PCOS have shown improvements in body composition (Aye et al., 2018; Costa et al., 2018; Kirk et al., 2019; dos Santos et al., 2020), insulin sensitivity (Al-Eisa et al., 2017; Aye et al., 2018; Kirk et al., 2019) and hormonal profile (Al-Eisa et al., 2017; Aye et al., 2018). Thus, international guidelines recommend that individuals with PCOS achieve 150-mins of MISS exercise, or 75-mins of vigorous-intensity activity per week (Teede et al., 2018). However, these PCOS-specific guidelines are based on general population data due to a lack of high-quality controlled trials in this population (Stepto et al., 2019). Consequently, the optimum exercise prescription for the management of PCOS is currently unknown.

Emerging data suggest that high-intensity interval exercise (HIIT) may improve cardiometabolic risk factors in individuals with PCOS and may improve exercise adherence (Almenning et al., 2015; Greenwood et al., 2016). However, interpretation of these data is hampered by inconsistency in the interventions utilized, incorporation of diet and/or pharmacological interventions, widely varied modalities, intensities and prescriptions, and small participant numbers. It is therefore challenging to establish the true effects of HIIT on outcomes and thus its role in PCOS management (Stepto et al., 2019). The primary aim of this systematic review and meta-analysis was to establish the impact of both MISS and HIIT exercise interventions on cardiorespiratory fitness and insulin resistance. Our secondary aim was to investigate the influence of both prescriptions on anthropometric and lipid profiles.

## Methods

### Protocol and Registration

This meta-analysis was approved and registered with PROSPERO (registration number: CRD42021255461).

### Ethical Approval, Search Strategy and Data Extraction

**Figure 1:**
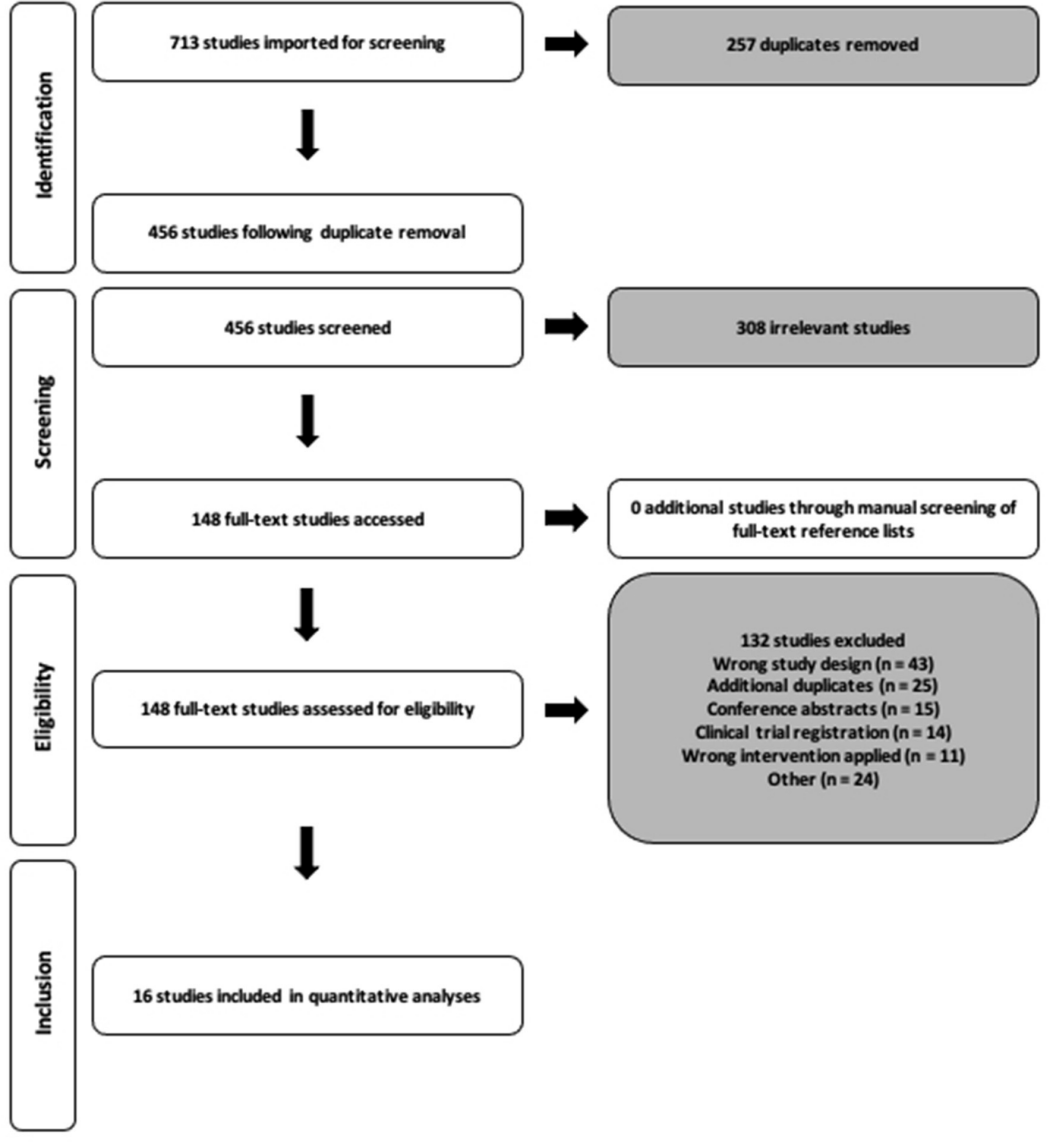
PRISMA study selection flow diagram.

We performed a systematic search of the literature in accordance with the Preferred Reporting Items for Systematic Reviews and Meta-Analyses (PRISMA) (Figure 1) of all publications up to 14th April 2021 utilizing the Pubmed, Scopus, EBSCO and ovidMEDLINE databases. Search terms were modified when required for the purpose of each database and consisted of the terms *Polycystic Ovary syndrome, exercise, fitness, insulin, body mass index and hyperandrogenism* (Supplementary Material A). Restrictions on search limits where possible included research in humans, females and studies written in the English language. Following the removal of all duplicates, two reviewers (CTR and RNL) independently screened all identified titles and abstracts, and full texts. Any disagreements throughout this process were discussed and consensus reached by a third reviewer (VLM). The reference list of all included studies following full-text review were manually screened to identify any other potential studies to include within the analysis.

Two authors (CTR and RNL) completed the extraction of all relevant data from eligible studies. Where reported, anthropometric, lipid profile, cardiometabolic profile, cardiorespiratory fitness data and sample sizes were extracted through the Covidence software (Covidence, RRID:SCR_016484, version 1) into a predesigned form (Microsoft Excel, RRID:SCR_016137, version 16.49). Where a trial produced multiple publications, results were merged and the largest participant number for each outcome was used in the quantitative synthesis. Where data were unclear or unable to be extracted as presented in the manuscript, the authors were contacted via email twice. If no response was received within 14 days of the second email, or raw data was unable to be provided, the study was excluded from the meta-analysis.

### Participants, Eligibility and Interventions

Utilizing the Participant, Intervention, Comparison, Outcome and Studies framework, our systematic review consisted of females diagnosed with PCOS through any recognized criteria between the ages of 18–50 years (Table 1). Inclusion in the meta-analysis was under the premise that the participants completed an isolated HIIT or MISS exercise intervention that did not include any concurrent treatment, including dietary manipulation, drug interventions or resistance training. Control data were collected from eligible studies that utilized PCOS controls who stated the following: participants were not provided with an exercise intervention; participants were not eligible to participate if they were exercising more than twice per week; were told to maintain their normal lifestyle with no change; and/or continue with standard care offered by their GP. Odds ratios (OR) comparing exercise vs. usual care for primary outcomes were calculated. The British Association for Cardiovascular Prevention and Rehabilitation (BACPR, 2019) guidelines were utilized for the categorization of MISS [40–70% $\dot{\text{V}}$O_2max_ /heart rate reserve (HRR) or 60–80% maximal heart rate (HRmax)]. HIIT was defined as exercise that was repetitive and intermittent in nature, with intensities exceeding 90% HRmax, 85%HRR or 85% $\dot{\text{V}}$O_2max_, in accordance with Norton et al. (2010). Exercise interventions that began within one threshold and traversed into another during the intervention period were excluded.

**Table 1:** Population, interventions, comparators, outcomes and study designs framework.

**Participant**	**Intervention**	**Comparison**	**Outcome**
PCOS diagnosis by any established criteria	Exercise intervention of moderate-intensity exercise (40–70% $\dot{\text{V}}$O_2max_/%HRR or 60–80% HR_max_) or high-intensity exercise (repetitive bouts of exercise above the maximum threshold of moderate intensity exercise, interspersed by active/rest periods)	HIIT vs. MISS	Primary: Insulin resistance (HOMA-IR) and cardiorespiratory fitness ($\dot{\text{V}}$O_2max_)
Premenopausal women aged 18–50			Secondary: anthropometrics (body mass, BMI and WC), lipid profile (HDL-C, LDL-C, TC and triglycerides), fasting insulin and fasting glucose
No weight restrictions			

*PCOS, polycystic ovary syndrome; HRR, heart rate reserve; HR_max_, maximal heart rate; HIIT, high-intensity interval training; MISS, moderate-intensity interval training; BMI, body mass index; WC, waist circumference; HDL-C, high-density lipoprotein cholesterol; LDL-C, low-density lipoprotein cholesterol; TC, total cholesterol*.

The primary outcomes of the meta-analysis were measures of insulin resistance through homeostatic model assessment of insulin resistance (HOMA-IR) and cardiorespiratory fitness, as measured by relative maximal oxygen consumption ($\dot{\text{V}}$O_2max_). Secondary outcomes included anthropometrics (body mass, body mass index [BMI] and waist circumference), cardiometabolic indices such as lipid profile (high-density lipoprotein cholesterol [HDL-C], low-density lipoprotein cholesterol [LDL-C], total cholesterol [TC] and triglycerides), fasting glucose and fasting insulin. Data on exercise adherence and fidelity was extracted from studies where provided. Adherence was calculated as the number of sessions completed divided by the number of sessions prescribed. Exercise fidelity was reported as a % achievement of target exercise intensity.

### Data Analysis

The mean ± standard deviation (SD) and sample size were input for each variable where provided. Where standard error of the mean (SEM) was presented, SD was calculated by:

$$\begin{array}{l} SEM\;x\;\sqrt{}N. \end{array}$$

where 95% confidence intervals (CI) were presented, the SD was calculated by:

$$\begin{array}{l} \sqrt{}N\;x\;\left(Upper\;limit\;of\;CI\;Lower\;limit\;of\;CI\right)/3.92 \end{array}$$

All outcome variables were input into the analysis software (Comprehensive Meta-analysis software (V.2.0), Biostat, Englewood, NJ, USA). To establish the effect of exercise in PCOS compared with usual care non-exercising PCOS controls, random effects OR were calculated on primary outcomes for HIIT and MISS exercise interventions, and reported as [OR (95% CI = lower [lwr.] to upper [upp.]), p = x]. Random-effects meta-analyses were run on each individual outcome variable in order to account for heterogeneity within the sample. The random-effects model provides a buffer for the individual variation that is inevitable between studies due to effect sizes and sample variation, and allows for a more comparable estimate of the true effect. Using the DerSimonian and Laird (1986) method, weighted means (Δ), standard error (SE), variance, and 95% confidence intervals (CI; lwr. to upp.) were calculated for each outcome variable and reported as [Δ, (95% CI = lwr. to upp.), p = x]. Forest plots of the standard difference in means ± 95% confidence intervals were created for each individual meta-analysis. Analyses were grouped to allow comparisons between the impact of HIIT exercise vs. MISS exercise interventions on each individual outcome variable.

### Risk of Bias and Quality of Evidence Assessment

Publication bias was assessed through funnel plots on primary outcomes and was reported for grouped analyses. The weighted sum of squared differences between individual study effects and the pooled effect across the studies (Q), and the percentage of variation across the studies due to heterogeneity (I^2^) was reported as (Q, I^2^, p = x).To assess the quality and validity of the included studies, the Tool for the assEssment of STudy quality and reporting in EXercise (TESTEX) was utilized (Smart et al., 2015). This scale utilizes a points system of a maximum of 15 points awarded for quality and reporting, and is specialized for the use in exercise interventions. Studies scored between 0–5 were classified as low-quality evidence, between 6–10 as moderate-quality evidence, and 11–15 as high-quality evidence. Two authors (CTR and RNL) independently assessed study quality using the TESTEX checklist, and any conflicts were resolved by a third reviewer (VLM).

## Results

### Search Outcomes

The systematic search of the literature returned 713 studies (Figure 1). Following title/abstract and full-text screening, 16 studies were included within the final analysis. For the included 16 studies, the total sample size for our primary outcomes of HOMA-IR and $\dot{\text{V}}$O_2max_ was 219 (HIIT = 60; MISS = 159) and 222 (HIIT = 28; MISS = 194), respectively. The number of studies included in the individual analyses varied due to inconsistency of reported outcomes across the literature. Study quality score and characteristics, including intervention exercise prescription, adherence and fidelity are reported in Table 2.

**Table 2:** Study characteristics and quality assessment scores.

**Author**	**Participant characteristics (Age [years] and BMI [kg/m^**2**^])**	**Quality assessment score**	**PCOS diagnostic criteria**	**Exercise intervention**	**Modality**	**Duration of intervention**	**Session frequency**	**Exercise intensity**	**Session duration**	**Supervised**	**Adherence**	**Fidelity**
Al-Eisa et al. (2017)	Age = 27.9 ± 4.1:BMI = 33.5 ± 2.8	5	Rotterdam	MISS	Treadmill walking	12 weeks	3 × p/w	65–75% HRR	45 mins	Yes		
Almenning et al. (2015)	BMI = 23.8 ± 4.8	11	Rotterdam	HIIT	Treadmill or outdoor walking/running and/or cycling (self-selected)	10 weeks	3 × p/w	2 × (90–95% HR_max_) 1 × “maximal intensity”	2 × ~40 mins 1 × ~35 mins	Partial	80%	
Aye et al. (2018)	Age = 28.3 ± 6.5:BMI = 29.4 ± 25.5	7	Rotterdam	MISS	Treadmill	8 weeks	3 × p/w	60% $\dot{\text{V}}$O_2max_	60 mins	Yes		
Benham et al. (2021)	Age = 29.1 ± 4.1:BMI = 31.4 ± 8.6	9	Rotterdam	HIIT	Aerobic exercise equipment of choice	24 weeks	3 × p/w	10 × (30secs:90secs) @ 90% HRR or 9/10 Borg rating	~ 20–25 mins	Partial	81%	65% (51%, 85%)^*^
	Age = 29.5 ± 4.6:BMI = 31.2 ± 9.0			MISS	Aerobic exercise equipment of choice	24 weeks	3 × p/w	50–60% HRR or 4–6/10 Borg rating	40 mins	Partial	79%	81% (56%, 85%)^*^
Covington et al. (2016)	Age = 25.6 ± 3.1:BMI = 32.1 ± 5.2	6	Rotterdam	MISS	Treadmill walking/running	16 weeks	5 × p/w	55% $\dot{\text{V}}$O_2max_	~ 23 mins (W1-4) → ~ 58 mins (W13-16)	Yes		
Faryadian et al. (2019)	Age = 34.3 ± 4.7:BMI = 21.2 ± 1.7	4	Rotterdam	HIIT	Running	12 weeks	3 × p/w	2 × [4 × (4mins:3mins) @ 90–95% HR_max_] 1 × [10 × (1min:1min) @ “maximal intensity”]	2 × ~35–40 mins 1 × ~30–35 mins			
Giallauria et al. (2008)	Age = 22.8 ± 3.7:BMI = 29.2 ± 2.9	9	Rotterdam	MISS	Cycle ergometer	12 weeks	3 × p/w	60–70% $\dot{\text{V}}$O_2max_	40 mins	Yes	100%	67%
Jedel et al. (2011)	Age = 30.2 ± 4.7:BMI = 27.7 ± 6.4	7	Rotterdam	MISS	Self selected (walking/cycling/ aerobic exercise)	16 weeks	3 × p/w	HR above 120bpm	~ 30 mins	No	73%	
Kirthika et al. (2019)		6	Rotterdam	MISS	Treadmill	12 weeks	3 × p/w	6km/h	45 mins	Yes	93%	
Orio et al. (2016)	Age = 25.9 ± 2.7:BMI = 26.7 ± 2.8	8	Rotterdam	MISS	Cycle ergometer	24 weeks	3 × p/w	60–70% $\dot{\text{V}}$O_2max_	45 mins	Yes	78%	
Randeva et al. (2002)	Age = 29.7 ± 6.8:BMI = 34.0 ± 4.5	7	NIH	MISS	Walking	24 weeks	~ 3 × p/w	Above 120bpm	~20–60 mins	No		
Ribeiro et al. (2020)	Age = 29.0 ± 4.3:BMI = 28.7 ± 4.8	6	Rotterdam	HIIT	Treadmill	16 weeks	3 × p/w	70% HR_max_ → 85–90% HR_max_	30 mins → 50 mins	Yes	83%	97%
	Age = 29.1 ± 5.3:BMI = 28.4 ± 5.6			MISS	Treadmill	16 weeks	3 × p/w	65% HR_max_ → 75–80% HR_max_	30 mins → 50 mins	Yes	76%	85%
Roessler et al. (2013)	Age = 31.0 ± 4.9:BMI = 36.7 ± 4.7	7	Rotterdam	HIIT	Cycle ergometer and walking/running	8 weeks (2 week ramp)	3 × p/w	(Cycle) 20s−3 mins work @ 80–100% HR_max_ : 25s−3 mins rest @ 45–65% HR_max_ (Walking/Running) 3–5 mins work @ 80–90% HR_max_ : 1 min rest @ 50–60% HR_max_	55 mins		82%	67%
Sprung et al. (2013)	Age = 28.0 ± 4.8:BMI = 33.0 ± 3.2	6	Rotterdam	MISS	NA	16 weeks	3 × p/w → 5 × p/w	30% HRR → 60% HRR	30 mins → 45 mins	Yes	100%	91%
Tiwari et al. (2019)	Age = 24.5 ± 4.8:BMI = 26.3 ± 3.7	9	Rotterdam	MISS	Marching	24 weeks	3 × p/w	HR above 120 bpm	30 mins	Partial		
Wu et al. (2021)	Age = 32.7 ± 3.2:BMI = 23.8 ± 3.0	6	Rotterdam	MISS	Cycle ergometer	12 weeks	4 × p/w	Individualized $\dot{\text{V}}$O_2AT_	60 mins	Yes		

* *Data were reported as median and IQR*.

*HR, heart rate; HRR, heart rate reserve*.

*Progression throughout the intervention is depicted by → *.

### High-Intensity Exercise Interventions

A total of five publications utilized HIIT as their method of intervention. Intervention duration ranged from 8 to 24 weeks (14.0 ± 6.3) with a session frequency of 3 sessions per week. Exercise modality varied between cycle ergometer (Roessler et al., 2013) treadmill walking/running and outdoor walking/running (Roessler et al., 2013; Faryadian et al., 2019; Ribeiro et al., 2020). Two studies (Almenning et al., 2015; Benham et al., 2021) allowed for participants to select their desired aerobic equipment to complete the exercise. Two studies reported partial supervision of the exercise intervention (at least 1 session supervised) (Almenning et al., 2015; Benham et al., 2021), one reported full supervision (Ribeiro et al., 2020) and two studies did not report supervision status (Roessler et al., 2013; Faryadian et al., 2019). Exercise intensity was prescribed using HR_max_ in four studies (Roessler et al., 2013; Almenning et al., 2015; Faryadian et al., 2019; Ribeiro et al., 2020), and HRR was utilized in a single study (Benham et al., 2021). Session duration ranged between 20 and 55 mins. Adherence to HIIT across these studies was 82 ± 1% and exercise fidelity was 82 ± 21%.

### Moderate-Intensity Exercise Interventions

A total of 12 publications utilized MISS as their method of exercise intervention. Intervention duration ranged from 8 to 24 weeks (16.6 ± 5.6), with session frequency ranging from 3 to 5 sessions per week. Exercise modality across MISS interventions varied, with cycle ergometer (Giallauria et al., 2008; Orio et al., 2016; Wu et al., 2021) and treadmill (Covington et al., 2016; Al-Eisa et al., 2017; Aye et al., 2018; Kirthika et al., 2019; Ribeiro et al., 2020) most frequently utilized. Two studies allowed participants to select their desired modality to complete the exercise (Jedel et al., 2011; Benham et al., 2021), while one reported using marching on the spot (Tiwari et al., 2019) and one did not report modality (Sprung et al., 2013). Eight studies indicated full supervision of the exercise intervention (Giallauria et al., 2008; Sprung et al., 2013; Covington et al., 2016; Orio et al., 2016; Aye et al., 2018; Kirthika et al., 2019; Wu et al., 2021), two studies indicated partial supervision (Tiwari et al., 2019; Benham et al., 2021) and two studies indicated no formal supervision (Randeva et al., 2002; Jedel et al., 2011). Exercise intensity was prescribed using %$\dot{\text{V}}$O_2max_ in four studies (Giallauria et al., 2008; Covington et al., 2016; Orio et al., 2016; Aye et al., 2018), HRR in three studies (Sprung et al., 2013; Al-Eisa et al., 2017; Benham et al., 2021) and %HR_max_ in one study (Ribeiro et al., 2020). Two studies utilized a minimum working heart rate of 120 bpm (Randeva et al., 2002; Tiwari et al., 2019), one study using a set treadmill speed of 6 km/h (Kirthika et al., 2019) and one used the individuals $\dot{\text{V}}$O_2_ achieved at anaerobic threshold ($\dot{\text{V}}$O_2AT_) (Wu et al., 2021). Session duration across the MISS intervention ranged from 20 to 60 mins. Adherence to MISS was 67 ± 9% and exercise fidelity was 86 ± 14%.

### Publication Bias

**Figure 2:**
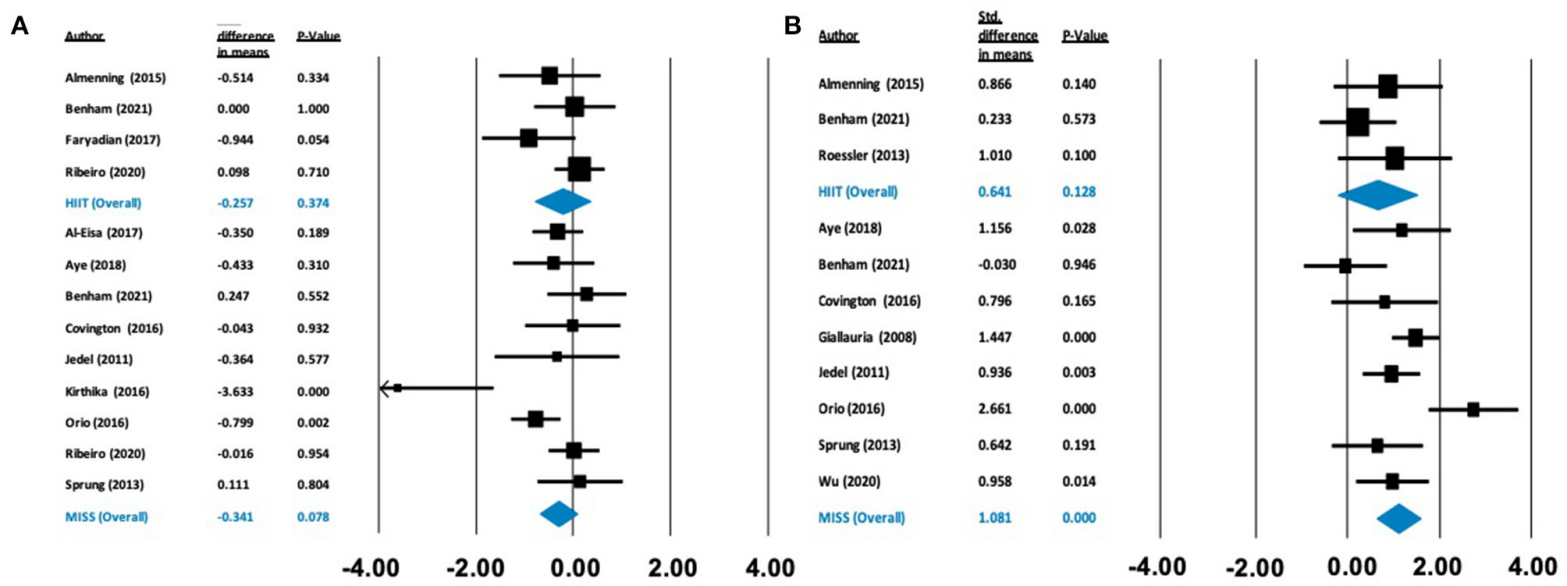
Forest plots of standard difference in means 95% ± confidence intervals for the effect of high-intensity interval training (HIIT) and moderate intensity steady state exercise (MISS) on **(A)** HOMA-IR as a measure of insulin resistance, and **(B)** maximal oxygen consumption (VO_2max_) as a measure of cardiorespiratory fitness, in polycystic ovary syndrome. Filled squares represent study outputs. Lines represent 95% confidence intervals. Filled blue diamonds represent the weighted mean determined through meta-analyses.

**Figure 3:**
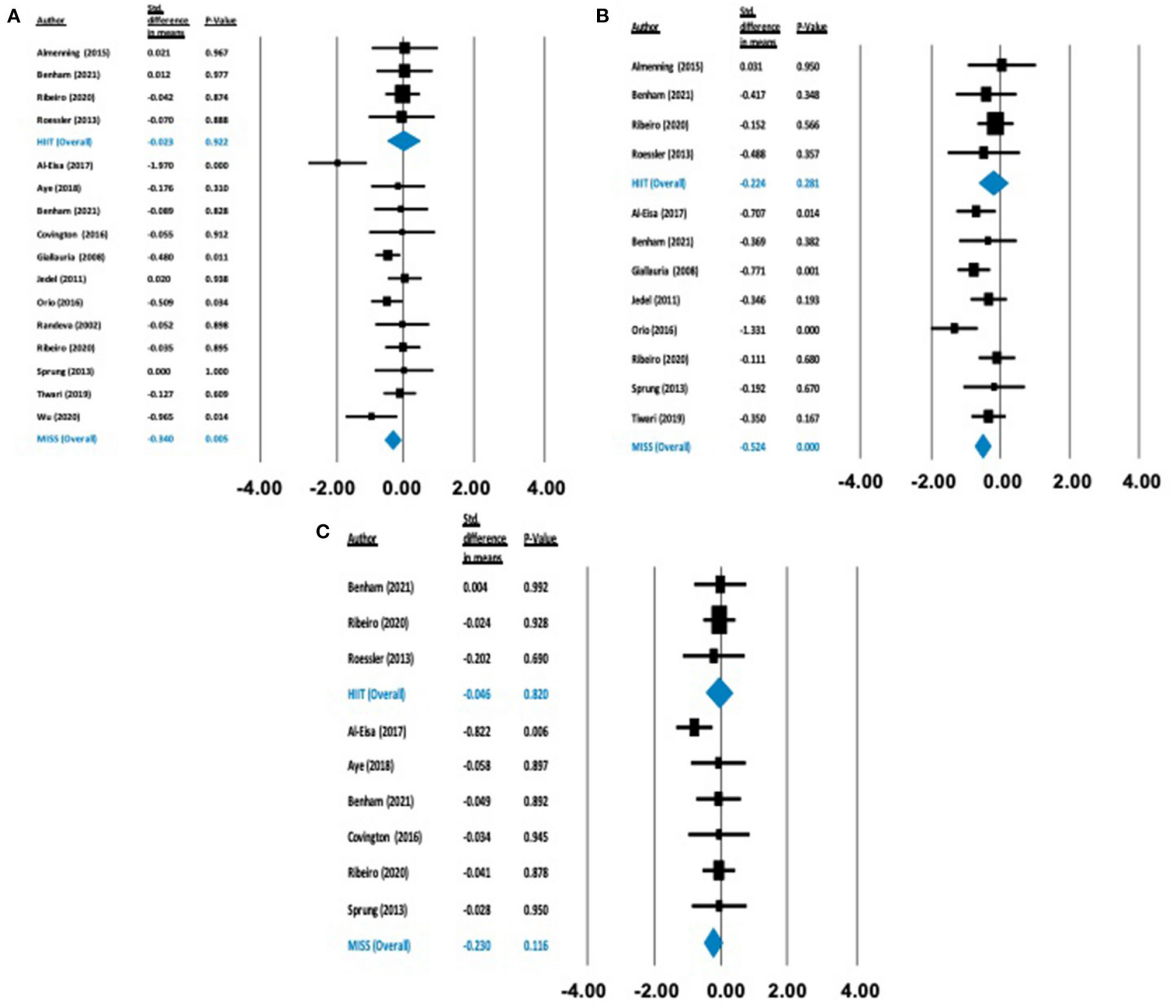
Forest plots of standard difference in means 95% ± confidence intervals for: **(A)** BMI, **(B)** waist circumference, and **(C)** body mass as measures of anthropometric profile. Filled squares represent study outputs. Lines represent 95% confidence intervals. Filled blue diamonds represent the weighted mean determined through meta-analyses.

**Figure 4:**
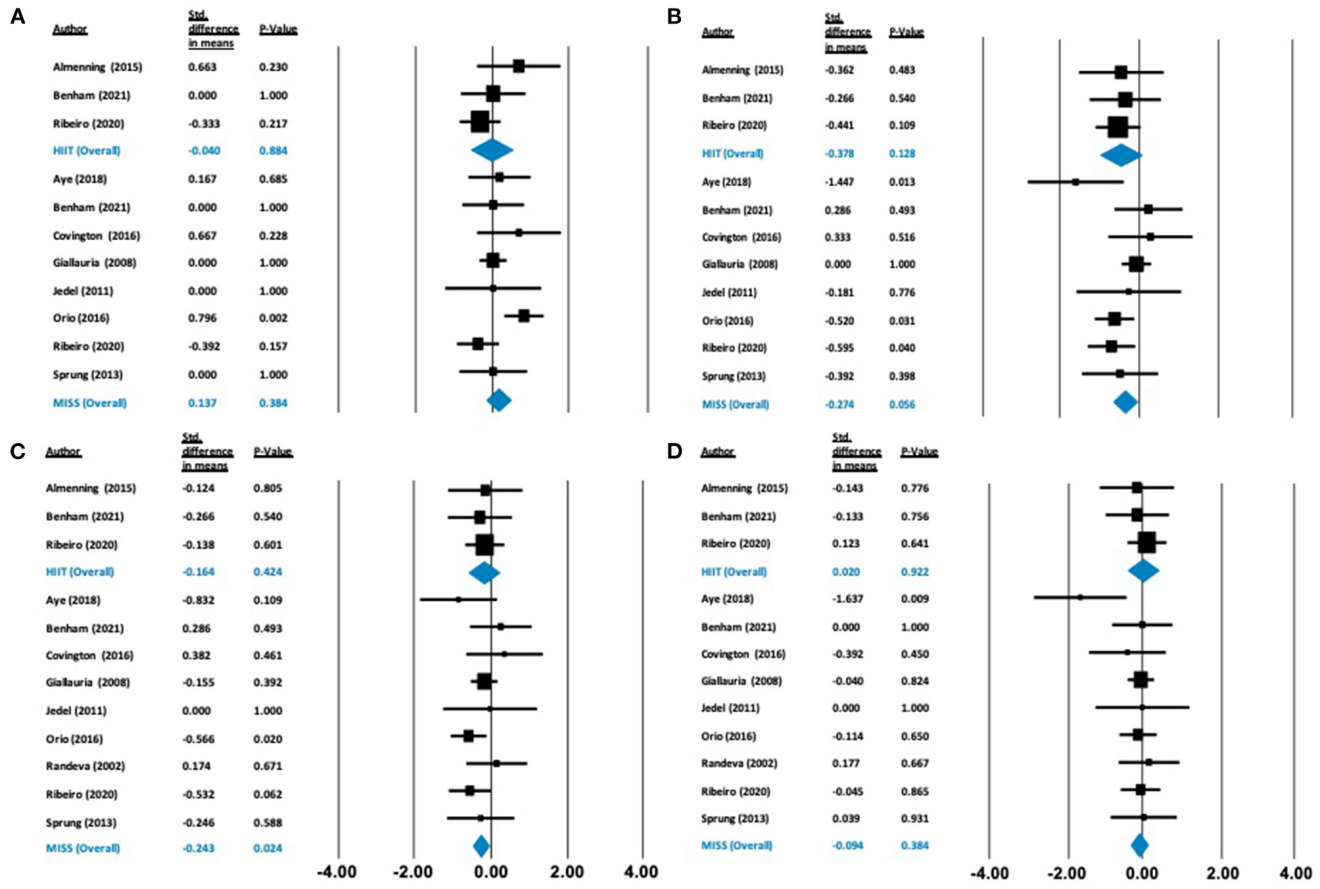
Forest plots of standard difference in means 95% ± confidence intervals for: **(A)** HDL-C, **(B)** LDL-C, **(C)** T-C, and **(D)** triglycerides as measures of lipid profile. Filled squares represent study outputs. Lines represent 95% confidence intervals. Filled blue diamonds represent the weighted mean determined through meta-analyses.

**Figure 5:**
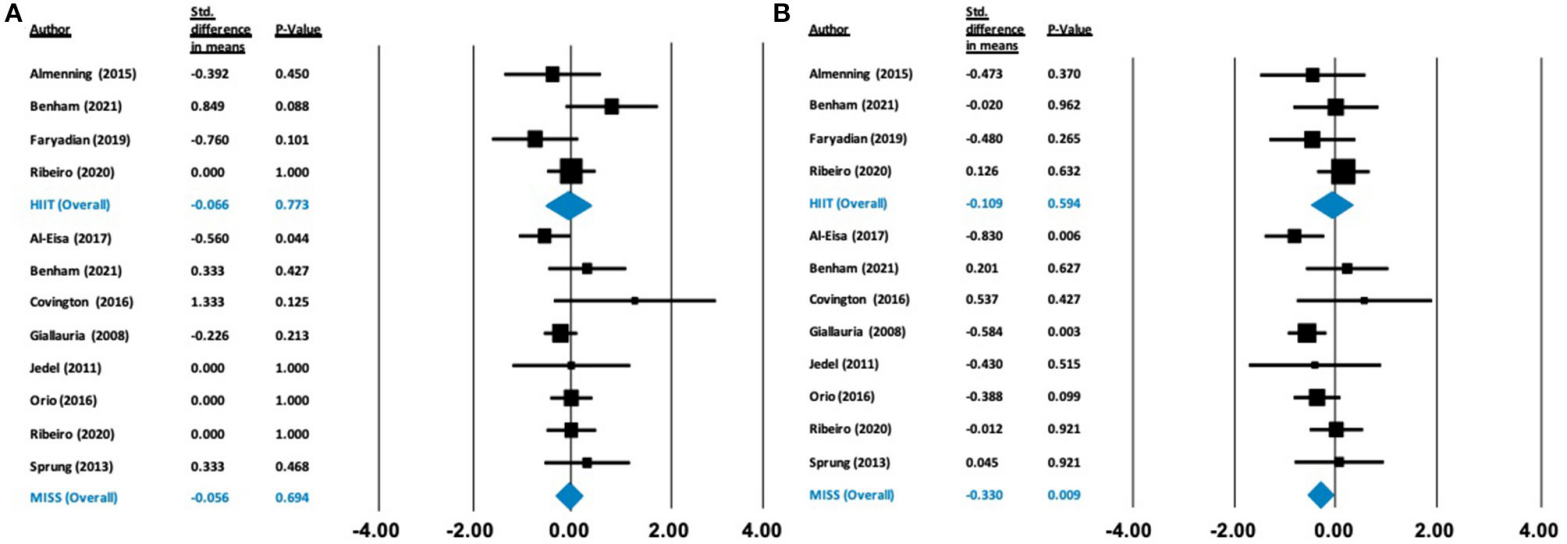
Forest plots of standard difference in means 95% ± confidence intervals for: **(A)** fasting glucose, **(B)** fasting insulin as measures of cardiometabolic profile. Filled squares represent study outputs. Lines represent 95% confidence intervals. Filled blue diamonds represent the weighted mean determined through meta-analyses.

There was significant heterogeneity in overall reported $\dot{\text{V}}$O_2max_ scores (Q = 24.43, I^2^ = 59%, *p* = 0.007) which, when grouped for HIIT (Q = 1.43, I^2^ = 0%, p = 0.490) and MISS (Q = 20.22, I^2^ = 65%, *p* = 0.005), was only evident in the MISS studies. There was also significant heterogeneity in overall reported HOMA-IR scores (Q = 23.39, I^2^ = 49%, *p* = 0.025), which was evidenced in only the MISS (Q = 18.71, I^2^ = 57%, *p* = 0.017) studies when grouped for exercise type (HIIT, Q = 4.10, I^2^ = 27%, *p* = 0.251). Analyses were not corrected for publication bias, and are shown below (Figures 2–5).

### Quality of Evidence

The evidence was rated as moderate-quality. Two studies (Al-Eisa et al., 2017; Faryadian et al., 2019) were of low-quality evidence, 13 studies (Randeva et al., 2002; Giallauria et al., 2008; Jedel et al., 2011; Roessler et al., 2013; Sprung et al., 2013; Covington et al., 2016; Orio et al., 2016; Aye et al., 2018; Kirthika et al., 2019; Tiwari et al., 2019; Ribeiro et al., 2020; Benham et al., 2021; Wu et al., 2021) were of moderate-quality evidence, and a single study (Almenning et al., 2015) was of high-quality evidence.

### Meta-Analyses

#### Odds Ratios

HIIT exercise did not statistically reduce HOMA-IR [1.641, (0.86–3.12), *p* = 0.131] or increase $\dot{\text{V}}$O_2max_ [1.899, (0.34–10.66), *p* = 0.466] compared with PCOS controls. Conversely, MISS exercise statistically, significantly reduced HOMA-IR [(1.727, 1.04–2.87), *p* = 0.035] and statistically increased $\dot{\text{V}}$O_2max_ [4.683, (1.92 – 11.43), *p* = 0.001] compared with PCOS controls [Supplementary Figures B and C (https://doi.org/10.6084/m9.figshare.c.5437518)].

#### Primary Outcomes

There was no effect on HOMA-IR following HIIT [Δ = −0.257 (−0.822 to 0.309), *p* = 0.374] or MISS exercise [Δ = −0.341 (−0.721 to 0.038), *p* = 0.078]. In contrast, there was a statistically significant increase in $\dot{\text{V}}$O_2max_ following MISS exercise [Δ = 1.081 ml/kg/min (0.624–1.537), *p* < 0.001], but not following HIIT [Δ = 0.641 ml/kg/min (−0.185 to 1.466), *p* = 0.128] (see Figure 2).

#### Anthropometric Outcomes

There was no statistically significant effect on BMI [Δ = −0.026 kg/m^2^ (−0.397 to 0.344), *p* = 0.890], body mass [Δ = −0.046 kg (−0.447 to 0.354), *p* = 0.820] or waist circumference [Δ = −0.224 cm (−0.634 to 0.183), *p* = 0.281] following HIIT exercise. Conversely, following MISS exercise, there were statistically significant reductions in BMI [Δ = −0.332 kg/m^2^ (−0.505 to −0.160), *p* = 0.000] and waist circumference [Δ = −0.524cm (−0.751 to −0.297), *p* = 0.000]. There was no statistically significant effect of MISS exercise [Δ = −0.230 kg (−0.517 to 0.057), *p* = 0.116] on body mass (Figure 3).

#### Cardiometabolic Outcomes

Our analysis showed no effect of either HIIT or MISS exercise on HDL-C ([Δ = −0.040 mmol/L (−0.574 to 0.495), *p* = 0.884] and [Δ = −0.137 mmol/L (−0.172 to 0.447), *p* = 0.384], respectively), LDL-C ([Δ = −0.378 mmol/L (−0.864 to 0.108), *p* = 0.128] and [Δ = −0.274 mmol/L (−0.555 to 0.007), *p* = 0.056], respectively) or triglycerides ([Δ = 0.020 mmol/L (−0.382 to 0.422), *p* = 0.922; and Δ = −0.094 mmol/L (−0.304 to 0.117), *p* = 0.384, respectively]) (Figure 4). Total cholesterol was not impacted by HIIT [Δ = −0.164 mmol/L (−0.567 to 0.239), *p* = 0.424], but was statistically, significantly reduced following MISS exercise [Δ = −0.243 mmol/L (−0.454 to −0.032), *p* = 0.024]. Fasting glucose remained unchanged following both HIIT [Δ = −0.066 mmol/L (−0.518 to 0.385), *p* = 0.773] and MISS exercise [Δ = −0.056 mmol/L (−0.332 to 0.221), *p* = 0.694] (Figure 5). In contrast, there was a statistically significant reduction in fasting insulin following MISS exercise [Δ = −0.330 pmol/L (−0.577 to −0.083), *p* = 0.009] but not HIIT [Δ = −0.019 pmol/L (−0.510 to 0.292), *p* = 0.594] (Figure 5).

## Discussion

### Summary of Main Findings

The aim of this analysis was to determine the effects of an isolated exercise intervention of HIIT or MISS exercise on measures of cardiorespiratory fitness and insulin resistance in individuals with PCOS from previously published data. We also sought to investigate the impact of HIIT and MISS exercise on anthropometric and cardiometabolic indices. The key findings from this analysis are (1) Only MISS exercise interventions improved $\dot{\text{V}}$O_2max_, (2) Neither exercise type improved HOMA-IR, (3) Only MISS exercise improved anthropometric profile, and (4) MISS exercise interventions decreased TC, but neither exercise type had any effect on HDL-C, LDL-C or triglycerides. Based on our analyses of the current moderate-quality evidence, MISS exercise appears to be a superior approach in improving cardiorespiratory fitness and BMI in women with PCOS, and should be prescribed as part of the comprehensive package of care for this condition. However, there is not enough high-quality evidence to disregard HIIT as a potential method of management of the condition, and further research is needed to understand the impact of HIIT exercise on outcomes in PCOS.

### Insulin Resistance

Insulin resistance is a common feature of PCOS independent of overweight or obesity (Burghen et al., 1980; Dunaif, 1997; Stepto et al., 2013), which can interplay with and exacerbate symptoms of the condition (Teede et al., 2007; Stepto et al., 2013). In our analysis, neither HITT nor MISS significantly improved HOMA-IR. Similar results have been reported recently, with no improvement in HOMA-IR or fasting insulin following 16 weeks of HIIT exercise (Lionett et al., 2021) and equivocal results following MISS exercise (Shele et al., 2020) in individuals with PCOS. However, we did observe a significant reduction in fasting insulin following MISS exercise, which may suggest improved insulin sensitivity, as a reduced amount of insulin is required to act upon a given concentration of glucose in order to maintain normal metabolic homeostasis (Iaccarino et al., 2021). One potential mechanism that may underpin the differences that appear within our analysis is a shift toward more oxidative and insulin-sensitive fiber type (T_1_) in the skeletal muscle (Wojtaszewski and Richter, 2006; Fisher et al., 2017). Longer duration, moderate-intensity, aerobic-based exercise, but not HIIT, has been associated with an increased percentage of T_1_ fibers (Wilson et al., 2012). Human and rodent studies (Fisher et al., 2017) have suggested that a greater insulin-stimulated glucose uptake in T_1_ muscle fibers is related to insulin sensitivity, therefore increased T_1_ muscle fibers may improve metabolic health.

A recent review has suggested that exercise volume may also play a pivotal role in controlling insulin sensitivity (Iaccarino et al., 2021). The authors reported that exercise interventions of ~ 170 mins of weekly exercise showed greater improvements in insulin sensitivity than interventions of ~115 mins/week. From our synthesis, the mean weekly exercise MISS interventions was around the 170 minute threshold (164 ± 59 mins/week), whereas the HIIT interventions did not meet this threshold (124 ± 31 mins/week). This may explain the improvement shown in fasting insulin following MISS interventions in this cohort compared with HIIT, and may indicate that individuals with PCOS should complete a larger volume of exercise if their aim is improving insulin sensitivity. It is also important to note that improvements in insulin sensitivity can be lost within 4 days of exercise cessation independent of exercise type (Ryan et al., 2020). Therefore, the timing of any post-intervention assessments may also explain the lack of change in HOMA-IR seen in our analysis. In addition, studies included within our analysis used HOMA-IR to measure insulin sensitivity. The euglycaemic–hyperinsulinaemic clamp is the gold standard measure of insulin sensitivity in humans and is more sensitive to small fluctuations in insulin sensitivity compared to HOMA-IR (Muniyappa et al., 2008). This may explain the lack of change in insulin sensitivity following both MISS and HIIT interventions, however few studies report insulin sensitivity using the euglycaemic–hyperinsulinaemic clamp method, and HOMA-IR is commonly used as the clinical measure of insulin sensitivity (Muniyappa et al., 2008).

### Cardiorespiratory Fitness

An increase in $\dot{\text{V}}$O_2max_ of 1-MET (equating to an ~3.5 ml/kg/min increase in oxygen consumption) can reduce the risk of CVD related mortality by 15% (Kodama et al., 2009). Our synthesis suggests that MISS exercise significantly improves $\dot{\text{V}}$O_2max_ by ~3 ml/kg/min, equating to an ~11% risk reduction for all-cause mortality (Kodama et al., 2009). MISS exercise also resulted in an increase in $\dot{\text{V}}$O_2max_ four-fold greater than usual care, non-exercising PCOS controls. These increases were evident in relative $\dot{\text{V}}$O_2max_ and are unlikely due to changes in body mass. This therefore likely reflects an improvement in absolute $\dot{\text{V}}$O_2max_ rather than a change in body composition. Surprisingly, these significant improvements were absent following HIIT, despite a mean improvement of ~2.8 ml/kg/min, which would confer similar reductions in mortality risk (Kodama et al., 2009). Furthermore, differences in $\dot{\text{V}}$O_2max_ outcomes may be attributed to the total volume of exercise stimulus that participants were exposed to. Our analysis showed that MISS interventions were 2.6 weeks longer in duration and included 14 more exercise sessions during the intervention period than those partaking in HIIT exercise interventions. This shorter study duration may have limited the improvements in $\dot{\text{V}}$O_2max_ in the HIIT interventions. In addition, of the HIIT interventions assessed, two studies (Roessler et al., 2013; Benham et al., 2021) reported exercise fidelity of ~65%. The inability to achieve the desired intensity within these interventions may also explain the lack of significant improvement in the HIIT interventions.

Our results deviate from previous studies where significant improvements in $\dot{\text{V}}$O_2max_ were evident following HIIT interventions in obesity (Chin et al., 2020), cardiometabolic disease (de Nardi et al., 2018; Boff et al., 2019) and PCOS (Lionett et al., 2021). This deviation may be a result of a significant publication bias toward studies reporting increases in $\dot{\text{V}}$O_2max_ in the MISS literature which was not evident within the HIIT literature included in our analyses. In addition, selection bias from participants may be impacting the improvement in $\dot{\text{V}}$O_2max_. The HIIT participants in our analysis had a higher baseline $\dot{\text{V}}$O_2max_ (30.3 ± 4.9 ml/kg/min) compared with the MISS group (26.3 ± 4.6 ml/kg/min). Exercise interventions typically result in the greatest improvement in $\dot{\text{V}}$O_2max_ in those with the lowest baseline values. Therefore, the lack of improvement in $\dot{\text{V}}$O_2max_ following HIIT interventions may be explained by baseline differences in cardiorespiratory fitness between the HIIT and MISS cohorts in our analysis, the publication bias in the MISS studies included, or differences in the duration and frequency of exercise interventions employed.

Importantly, in our analysis, MISS interventions resulted in a significant improvement in $\dot{\text{V}}$O_2max._ Increased mitochondrial oxidative capacity is linearly correlated with improvements in $\dot{\text{V}}$O_2max_ (van der Zwaard et al., 2016). MISS exercise induces an increase in mitochondrial volume and density, and a subsequent increase in respiratory capacity of the mitochondria (Holloszy and Coyle, 1984), potentially mediated by a shift toward T_1_ skeletal muscle fibers. Increases in mitochondria are also only evident in T_1_ muscle fibers following 12 weeks of MISS exercise and are not evident following the same duration of sprint-interval training (Skelly et al., 2021). Taken together, this suggests that MISS exercise, but not HIIT exercise, may improve oxidative capacity in individuals with PCOS through changes in muscle fiber type and mitochondrial density.

### Anthropometric Profile

Overweight and obesity are prevalent in more than 50% of individuals with PCOS and have the potential to exacerbate symptoms of the condition (Diamanti-Kandarakis and Dunaif, 2012). Our analyses showed that MISS exercise induced significant reductions in BMI (−3.3 kg/m^2^) and waist circumference (−2.84 cm) which were absent following HIIT exercise interventions. These results are similar to previous studies, where there is a clearly established dose-response relationship between total exercise volume and reductions in weight (Slentz et al., 2004). Our analysis is also in line with previous work in healthy and diseased cohorts, where HIIT exercise has not been shown to elicit improvements in anthropometric profile, likely related to the lower exercise volume employed in HIIT interventions (Sultana et al., 2019; Viana et al., 2019). The observed reductions in waist circumference following MISS exercise may reflect important benefits for individuals with PCOS, as visceral adipose accumulation is associated with increased insulin resistance and systemic inflammation (Kojta et al., 2020), and an ~25% increased mortality risk, independent of BMI (Koster et al., 2008). Therefore, MISS exercise should be prescribed to individuals with PCOS as a means of reducing anthropometric indices, especially visceral adiposity, which may result in metabolic health benefits.

### Cardiometabolic Profile

Commonly, individuals with PCOS present with dyslipidaemia, characterized by reduced HDL-C, elevated triglycerides and increased LDL-C concentrations (Wild et al., 2011). LDL-C is established as a potent risk factor for the development of CVD (Ference et al., 2017). Lowering of LDL-C concentration through pharmacological intervention has been shown to reduce the risk of cardiovascular events (Ference et al., 2017; Johannesen et al., 2020). However, aerobic exercise alone does not appear to change LDL-C levels unless accompanied by weight loss (Katzmarzyk et al., 2001; Wang and Xu, 2017), and may not be sensitive to low-moderate intensity exercise (Albarrati et al., 2018). In accordance with this, our results showed no significant reduction in LDL-C following MISS or HIIT exercise alone, despite a significant decrease in weight following MISS. Longer-term (16 weeks) intervention has been shown to reduce LDL-C significantly following treatment with diet and exercise combined, with optimal reductions in LDL-C observed after 12 months (Varady and Jones, 2005). The mean duration of isolated exercise of both HIIT and MISS interventions were 14 and 16 weeks, respectively, neither of which induced significant change. Therefore, to reduce LDL-C, a combination of diet and exercise may be required over a longer-term duration.

Our findings following MISS exercise showed a significant reduction in TC, which incorporates both HDL-C and LDL-C and can be therefore be misleading. It is likely that a significant reduction in TC following MISS exercise can be attributed to the non-significant reduction in LDL-C given no impact of MISS exercise on HDL-C. A reduction in TC is important for long-term cardiovascular disease risk in this population, and has been previously associated with volume of exercise (Varady and Jones, 2005; Kodama et al., 2007). As exercise volume is lower in HIIT compared to MISS, this may also explain the improvement in TC evident following MISS interventions only. Intriguingly, HDL-C did not change as a result of either exercise type. Therefore, these results may also be due to the inclusion of PCOS patients with lipid profiles within normal ranges, who did not present with hyperlipidaemia. Our analysis investigated the impact of isolated exercise without concurrent intervention, such as diet or lifestyle modification. Kodama et al. (2007) suggest that exercise alone only improves HDL-C when MISS exercise at a volume (duration and frequency) greater than exercise guideline recommendations is employed. Exercise alone may also not improve HDL-C levels in those with a higher BMI (Kodama et al., 2007) and weight loss may need to accompany exercise to increase plasma HDL-C (Nicklas et al., 1997). Therefore, while MISS exercise may have some beneficial effect on cardiometabolic profile due to a greater overall exercise volume, patients with PCOS may require additional dietary and/or pharmacological interventions to appropriately control dyslipidaemia.

### Limitations

There was a significant publication bias in the analysis of MISS interventions that demands caution when interpreting these results. There are very few randomized controlled trials on the impact of exercise without concurrent intervention. Therefore, we were required to extract data from a wider range of methodological studies where there was potential for researcher bias, participant selection, small samples of participants and small study numbers. This also impacts on study quality, where most evidence synthesized was of moderate quality. Our review was also focused on the cardiometabolic aspects of PCOS and did not include analysis of androgen levels or clinical symptoms. Finally, despite PCOS having multiple phenotypes, only four studies (Jedel et al., 2011; Almenning et al., 2015; Ribeiro et al., 2020; Benham et al., 2021) explicitly reported phenotypical subgroups within their analyses. Previous data suggest that the different phenotypical presentations of PCOS may respond differently to exercise stimuli hence this is an important area for further study (Borzan et al., 2021).

### Future Direction

Our analysis highlights the requirement for larger, randomized controlled trials to be conducted in order to further our understanding of PCOS, and how exercise, especially HIIT, can be utilized as a tool for disease management. Such studies should include an analysis of androgen concentrations and clinical manifestations of the condition. Future exercise studies should also report on exercise adherence, compliance and fidelity of the programme in order to further understand the optimal method of exercise to help manage this condition, in addition to analyzing the impact of these factors on exercise behavior following the intervention period. Furthermore, the impact of different exercise modalities on PCOS phenotypes is required in order to discriminate any effect of PCOS sub-type, rather than employing a “one size fits all” approach.

## Conclusion

Our analysis is the first to compare the impact of isolated HIIT and MISS exercise intervention in individuals with PCOS. MISS exercise resulted in a four-fold increase in $\dot{\text{V}}$O_2max_ and significant reduction in HOMA-IR compared with controls receiving usual care from their GP. A beneficial impact of MISS exercise was also evident on anthropometric indices and total cholesterol in individuals with PCOS, which supports the value of MISS exercise prescription in disease management. In contrast, HIIT did not convey these benefits, although higher-quality evidence is required to fully understand the impact of HIIT on outcomes in PCOS before this can be excluded as a potential treatment option.

## Data Availability Statement

Publicly available datasets were analyzed in this study. This data can be found here: 10.6084/m9.figshare.14687526.

## Author Contributions

CR, DR, PJ, and RL conceived the study. CR acquired the data. CR, VM, and RL analyzed and interpreted the data. CR drafted the manuscript. All authors critically reviewed the manuscript. All authors provide final approval of the version to be published and agree to be accountable for the work.

## Conflict of Interest

The authors declare that the research was conducted in the absence of any commercial or financial relationships that could be construed as a potential conflict of interest.

## Publisher's Note

All claims expressed in this article are solely those of the authors and do not necessarily represent those of their affiliated organizations, or those of the publisher, the editors and the reviewers. Any product that may be evaluated in this article, or claim that may be made by its manufacturer, is not guaranteed or endorsed by the publisher.
