# Supplementary material for: HIIT'ing or MISS'ing the Optimal Management of Polycystic Ovary Syndrome: A Systematic Review and Meta-Analysis of High- Versus Moderate-Intensity Exercise Prescription
Source: Front Physiol. 2021 Aug 16;12:715881. doi: 10.3389/fphys.2021.715881 (PMC8415631; doi:10.3389/fphys.2021.715881)
Supplement: Supplementary file 1 [file Table_1.DOCX]

**Supplement A – Literature base search terms for multiple databases**

("Polycystic Ovary Syndrome"[Mesh])[Majr] AND "Exercise"[Majr] AND ("Insulin*" OR "Fitness" OR "Body Mass Index" OR "Hyperandrogenism") NOT "Review"

(Polycystic Ovary Syndrome [MeSH]) AND (Exercise/ or (exercise or physical* activ* or high intensity or HIIT or HIT or moderate intensity or MISS or aerobic or fitness))

S1 exercise or "physical* activ*" or "strenuous activit*" or "high intensit*" or HIT or “moderate intensit*” or mvpa or ltpa or "aerobic capacity" or fitness

TITLE-ABS-KEY ( ( "Polycystic Ovary Syndrome" )  AND  "Exercise"  AND  ( "Insulin*"  OR  "Fitness"  OR  "Body Mass Index"  OR  "Hyperandrogenism" ) )  AND  ( polycystic  AND ovary  AND syndrome )  AND  ( EXCLUDE ( DOCTYPE ,  "re" )  OR  EXCLUDE ( DOCTYPE ,  "cp" )  OR  EXCLUDE ( DOCTYPE ,  "ed" )  OR  EXCLUDE ( DOCTYPE ,  "sh" )  OR  EXCLUDE ( DOCTYPE ,  "ch" )  OR  EXCLUDE ( DOCTYPE ,  "le" )  OR  EXCLUDE ( DOCTYPE ,  "no" )  OR  EXCLUDE ( DOCTYPE ,  "tb" )  OR  EXCLUDE ( DOCTYPE ,  "bk" ) )  AND  ( LIMIT-TO ( EXACTKEYWORD ,  "Human" )  OR  LIMIT-TO ( EXACTKEYWORD ,  "Female" )  OR  LIMIT-TO ( EXACTKEYWORD ,  "PCOS" ) )

“Polycystic Ovary Syndrome” AND “Exercise” NOT “Review”

Limit: Human

(Exercise) AND (Polycystic Ovary Syndrome)

Limits: Interventional Studies, Female, Intervention/ Treatment – Exercise, Age – 18-64

TI, AB, KW “Polycystic Ovary Syndrome” AND TI, AB, KW “Exercise” AND TI,AB,KW (“Insulin” OR Body Mass Index” OR “Fitness” OR “Androgen*”
