# Supplementary material for: HIIT'ing or MISS'ing the Optimal Management of Polycystic Ovary Syndrome: A Systematic Review and Meta-Analysis of High- Versus Moderate-Intensity Exercise Prescription
Source: Front Physiol. 2021 Aug 16;12:715881. doi: 10.3389/fphys.2021.715881 (PMC8415631; doi:10.3389/fphys.2021.715881)
Supplement: Supplementary file 3 [file Table_3.DOCX]

Supplement C - Odds ratio for VO_2max_
